# Low ALT Is Associated with IBD and Disease Activity: Results from a Nationwide Study

**DOI:** 10.3390/jcm13071869

**Published:** 2024-03-24

**Authors:** Asher Shafrir, Lior H. Katz, Michal Shauly-Aharonov, Adar Zinger, Rifaat Safadi, Joshua Stokar, Itay Kalisky

**Affiliations:** 1 Meuhedet Health Medical Organization, Jerusalem District, Tel Aviv 6203854, Israel; 2 Institute of Gastroenterology and Hepatology, Hadassah Medical Center, Jerusalem 1200, Israel; klior@hadassah.org.il (L.H.K.); adar@hadassah.org.il (A.Z.); itayk@hadassah.org.il (I.K.); 3 Faculty of Medicine, Hebrew University of Jerusalem, Jerusalem 9190500, Israel; safadi@hadassah.org.il (R.S.); ystokar@hadassah.org.il (J.S.); 4 Braun School of Public Health and Community Medicine, Hebrew University of Jerusalem, Jerusalem 9190500, Israel; michal.shauly@mail.huji.ac.il; 5 The Jerusalem College of Technology, Jerusalem 9190401, Israel; 6 The Liver Institute, Hadassah Medical Organization, Jerusalem 91120, Israel; 7 Department of Endocrinology, Hadassah Medical Center, Faculty of Medicine, Hebrew University of Jerusalem, Jerusalem 9190500, Israel

**Keywords:** inflammatory bowel disease, alanine transaminase, sarcopenia, calprotectin

## Abstract

**Background**: Sarcopenia is underdiagnosed in patients with inflammatory bowel disease (IBD). Low alanine transaminase (ALT) is associated with sarcopenia. We evaluated the association between low ALT and the presence of IBD and disease activity. **Methods**: Data were collected from a national Israeli health insurer cohort comprising 976,615 patients. Patients with a diagnosis of IBD were compared to healthy controls. After exclusion of patients with liver disease, ALT > 40 IU/L and age < 18, a total of 233,451 patients were included in the analysis. Low ALT was defined as <10 IU/L. **Results**: Low ALT was more common amongst patients with IBD than in healthy controls (7.76% vs. 5.7% *p* < 0.001). Low ALT was found in 148 (7.9%) of the patients with CD and 69 (6.9%) of the patients with UC. For CD, low ALT was associated with increased fecal calprotectin (FC) and CRP (223.00 μg/mg [63.45–631.50] vs. 98.50 [31.98–324.00], *p* < 0.001, 9.10 mg/L [3.22–19.32] vs. 3.20 [1.30–8.30], *p* < 0.001) and decreased albumin and hemoglobin (3.90 g/dL [3.60–4.20] vs. 4.30 [4.00–4.50], *p* < 0.001,12.20 g/dL [11.47–13.00] vs. 13.60 [12.60–14.70], *p* < 0.001). For UC, low ALT was associated with higher FC and CRP (226.50 μg/mg [143.00–537.00] vs. 107.00 [40.85–499.50], *p* = 0.057, 4.50 mg/L [1.90–11.62] vs. 2.30 [1.00–6.20], *p* < 0.001) and with lower albumin and hemoglobin (4.00 g/dL [3.62–4.18] vs. 4.30 [4.10–4.40], *p* < 0.001, 12.40 g/dL [11.60–13.20] vs. 13.60 [12.60–14.60], *p* < 0.001). These findings remained consistent following multivariate regression and in a propensity score-matched cohort. **Conclusions**: Low ALT is more common in patients with IBD and is associated with biochemical disease activity indices.

## 1. Introduction

Inflammatory bowel disease (IBD), including ulcerative colitis (UC) and Crohn’s disease (CD), is characterized by chronic intestinal inflammation due to a complex interaction between genetic factors, disturbed epithelial barriers, uncontrolled inflammatory signals, loss of tolerance, and environmental triggers [1]. IBD is frequently complicated by malnutrition, defined as “a state resulting from lack of intake or uptake of nutrition that leads to altered body composition (decreased fat-free mass) and body cell mass leading to diminished physical and mental function and impaired clinical outcome from disease” [2]. Malnutrition is a crucial factor in the state of muscle loss known as sarcopenia [2]. Patients with quiescent disease have higher muscle mass than those with active bowel disease who are more likely to be malnourished and sarcopenic [3]. Even amongst patients with IBD who have a normal body mass index (BMI, calculated as kg/m^2^) and serum albumin level, body cell mass and handgrip strength are lower than in healthy controls. [4,5]. Sarcopenia is also associated with a higher risk of surgical complications [3,6,7]. Patients with IBD and sarcopenia are also more likely to suffer from osteopenia and osteoporosis [5]. A normal BMI cannot rule out the presence of sarcopenia, since a high percentage of patients with IBD are obese; thus, sarcopenia can be frequently underdiagnosed [8]. The clinical importance of sarcopenia warrants incorporating muscle strength when evaluating and tailoring management of IBD [9]. However, as many of these tests are time-consuming and are not available in many IBD centers, surrogate markers that could show patients at risk for malnutrition and sarcopenia are warranted.

Alanine aminotransferase [ALT, also known as serum glutamic-pyruvic transaminase (SGPT)] is a critical enzyme in the alanine cycle that is responsible for the transfer of the α-amino group from an α-amino acid to an α-keto acid, transforming pyruvate to alanine in skeletal muscle and catalyzing alanine to pyruvate in the liver [10]. Elevated ALT is a marker of liver damage as it is released to the blood by damaged hepatocytes [11]. Conversely, multiple studies have shown that low ALT is associated with increased mortality [12,13,14,15,16,17]. Low ALT is also associated with frailty, sarcopenia, and disability, which may all explain the increase in mortality [18,19,20]. Patients with lower skeletal muscle mass index have lower ALT than patients with a normal skeletal mass index [21]. Low ALT is also more prevalent in the geriatric population [18,22].

The association of IBD with elevated liver enzymes, including ALT, is well established [23] and in some studies has predicted complications and a worse prognosis [24,25]. Conversely, a higher prevalence of low ALT was reported in two small retrospective studies among pediatric and adult IBD patients [26,27]. However, data concerning the association of low liver enzymes, specifically ALT, with IBD are still scarce.

This study aims to explore the prevalence of low ALT amongst patients with IBD and the possible associations of low ALT with serum markers of disease activity in a nationwide health organization.

## 2. Methods

The Meuhedet health maintenance organization (HMO) is one of Israel’s four state-mandated HMOs. It serves over 1,300,000 individuals and is the third largest HMO in Israel. Meuhedet’s EMR (electronic medical record) includes real-time input from all physician visits, medical diagnoses, laboratory results, hospitalizations, and dispensing data on prescription and over-the-counter medications. Data of 976,615 patients were collected as part of a prospective study following all HMO patients who underwent SARS-CoV-2 testing from March 2020 to 31 December 2021. Data gathered included diagnoses documented in the EMR and blood tests in the year prior. As all data were from before any possible COVID-19 infection, there were no concerns regarding a confounding effect of COVID-19 on laboratory results. Based on ICD-9 coding, patients diagnosed with IBD were compared to a healthy controls. ALT levels were divided into three groups, low (ALT < 10 IU/L), normal (10–40 IU/L), and high (ALT > 40 IU/L). As high ALT levels point to liver disease, patients with ALT > 40 were excluded from the study [28].

Within the IBD cohort, various inflammatory and metabolic markers correlated with disease activity were compared between patients with low ALT with those with normal ALT. Patients with ICD-9 diagnoses of chronic liver disease, cirrhosis, a diagnosis of both CD and UC (indeterminate IBD), ALT > 40 IU/L, those who did not have an ALT level from the year prior to the study cohort, and those of age < 18 were excluded.

The study was conducted in accordance with the Declaration of Helsinki and approved by the research ethics committee and internal review board of Meuhedet HMO (02-24-08-20). Helsinki approval was granted on 2 September 2020.

### Statistical Analysis

For descriptive analyses, counts and percentages were used for categorical variables. Continuous normally distributed variables were summarized as means ± standard deviations (SD). Variables not normally distributed were summarized as medians with an interquartile rang. The Chi-squared test was used to compare categorical variables; Student’s *t*-test was used to compare means of normally distributed continuous variables; and the Wilcoxon rank test was used for variables not normally distributed. Multivariate logistic regression was performed to assess the association of IBD with low ALT while controlling for age, gender, smoking status, socioeconomic status (SES), and sector. The SES index is an integral part of the Meuhedet HMO electronic database and is provided by Points Location Intelligence (https://points.co.il/ (accessed on 4 March 2024)), being rated on a scale of 1–10. The index uses data that include average family size, income, educational level, unemployment rate, number of cars per family, and median age in the specific area in which the patient lives. Sector was categorized into Arab, ultra-orthodox Jewish, and orthodox/secular Jewish and is loosely identified based on the clinic’s location. For the analysis within the IBD cohort, multivariate linear and logistic regression models were constructed to evaluate the association between low ALT and different laboratory outcomes such as serum albumin, fecal calprotectin (FC), vitamin B12, ferritin, C reactive protein (CRP), and low vitamin D (>20 ng/mL). Additionally, 1:2 propensity score-matching (PSM) was used to compare patients based on age, sex, disease type, BMI, smoking, SES, and sector. Inflammatory and metabolic biomarkers were compared using a paired t-test or chi-square tests according to the compared variable. A *p*-value of less than 0.05 was considered statistically significant in all analyses. Statistical analysis was performed using R software version 3.6 (R Development Core Team, 2018).

## 3. Results

Starting from a total of 976,615 patients in the database, 233,451 patients remained after application of the exclusion criteria. Compared with patients with normal ALT, those with low ALT were younger (45.72 ± 22.84 vs. 46.90 ± 18.47, *p*-value < 0.001), more likely to be female (11,222 (83.8%) vs. 134,462 (61.1%), *p*-value < 0.001), and had a lowerBMI (25.62 ± 5.66 vs. 26.78 ± 5.41 *p*-value < 0.001). Additional comparisons are described in Table 1.

Low ALT was more common amongst patients with IBD (7.76% vs. 5.7% *p*-value < 0.001), which also remained statistically significant after application of multivariate logistic regression including age, gender, smoking, BMI, socioeconomic status, and sector (OR 1.51, 95% CI 1.29–1.76, *p*-value < 0.001).

Amongst patients with established IBD, 1869 (66.58%) had CD and 938 (33.44%) had UC. Amongst those with CD, 148 (7.9%) had ALT < 10, and 1721(92.1%) had normal ALT. The proportion of females was higher in the low-ALT group (111 (75.0%) vs. 898 (52.2%) *p*-value < 0.001), and BMI was lower (23.35 ± 4.81 vs. 25 ± 5.25, *p*-value < 0.001). Age and smoking were not significantly different between the two groups. Amongst patients with UC, 69 (6.9%) had low ALT and 930 (93.1%) had normal ALT. Amongst patients with UC, compared to those with normal ALT, patients with low ALT were younger (42.43 ± 19.53 vs. 48.98 ± 17.51, *p*-value = 0.003), more likely female (82.6% vs. 48.98%, *p*-value < 0.001), and had a lower BMI (24.19 ± 5.27 vs. 25.72 ± 4.77, *p*-value = 0.029). See Table 2 for additional comparisons.

### 3.1. Association with Inflammatory Biomarkers

**Figure 1:**
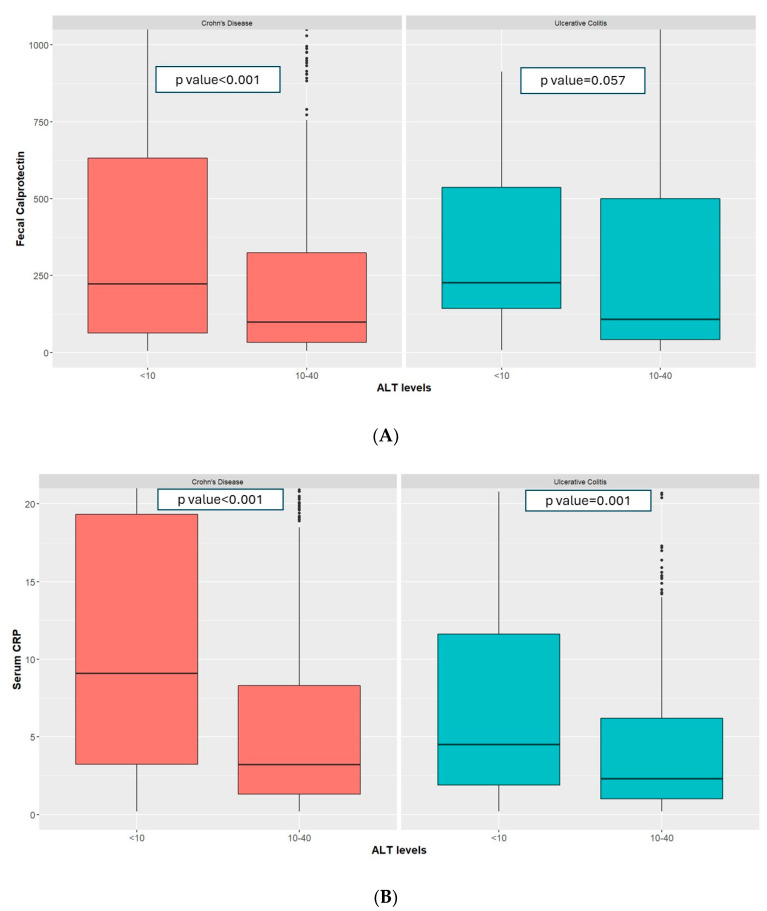
Fecal calprotectin (**A**) and CRP levels (**B**) amongst patients with UC and CD with low and normal ALT.

**Figure 2:**
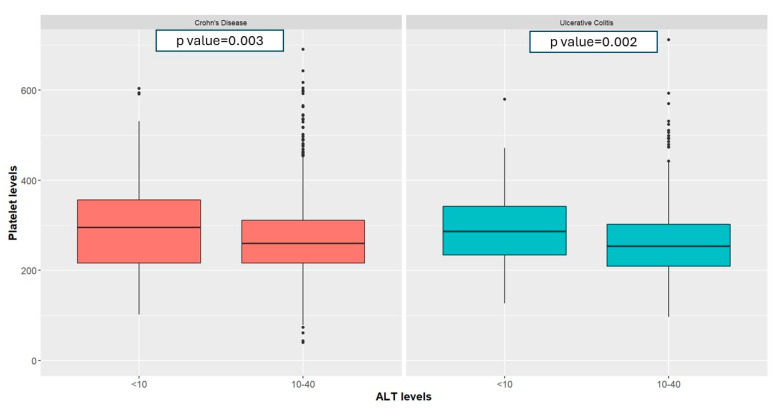
Platelet levels amongst patients with UC and CD with low and normal ALT.

Amongst patients with CD and low ALT, median fecal calprotectin was higher at 223.00 μg/mg [63.45–631.50] vs. 98.50 [31.98–324.00], *p*-value < 0.001, Figure 1A), as were CRP (9.10 mg/L [3.22–19.32] vs. 3.20 [1.30–8.30], *p*-value < 0.001, Figure 1B) and platelet levels (295,000 U/mL [215,750–350,000] vs. 260,000 U/mL [216,000–311,000], *p*-value = 0.003, Figure 2) compared to those with normal ALT. Additionally, the proportion of patients with elevated FC (>150 μg/mg) was higher in the low-ALT group (27.7% vs. 17.48%, *p*-value = 0.004). These findings were consistent when using a multivariate linear regression model controlling for age, gender, smoking status, BMI, socioeconomic status, sector, and IBD disease (Table 2).

In patients with UC, low ALT was associated with higher FC (226.50 μg/mg [143.00–537.00] vs. 107.00 [40.85–499.50], *p*-value = 0.057, Figure 1A), higher CRP (4.50 mg/L [1.90–11.62] vs. 2.30 [1.00–6.20], *p*-value < 0.001, Figure 1B), and a higher platelet count (286,000 U/mL [234,000–342,000] vs. 253,000 [209,000–302,000], *p*-value = 0.002, Figure 2). The proportion of patients with elevated FC (>150 μg/mg) was also higher (27.56% vs. 13.38%, *p*-value = 0.015) in patients with low ALT. These findings were consistent when using a multivariate linear regression model controlling for age, gender, smoking status, BMI, socioeconomic status, sector, and IBD disease (Table 2).

### 3.2. Association with Metabolic Markers

**Figure 3:**
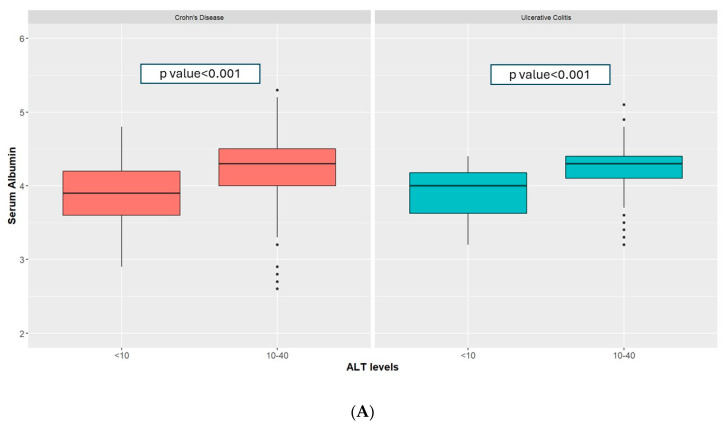

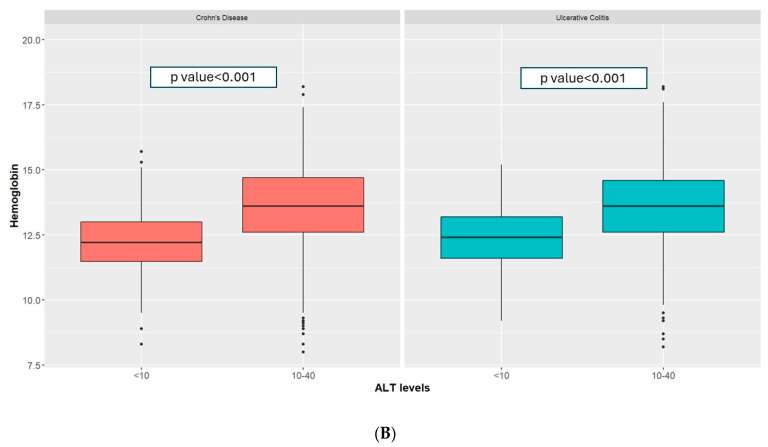
Serum albumin (**A**) and hemoglobin (**B**) amongst patients with UC and CD with low and normal ALT.

**Figure 4:**
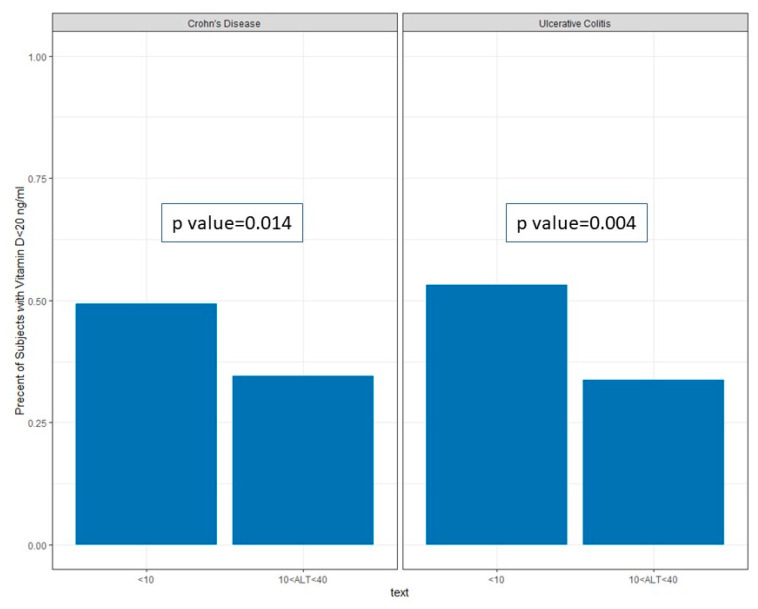
Proportion of patients with serum vitamin D levels < 20 ng/mL amongst low- and normal-ALT groups.

Amongst patients with CD and low ALT, serum albumin (3.90 g/dL [3.60–4.20] vs. 4.30 [4.00–4.50], *p*-value < 0.001, Figure 3A) and hemoglobin levels (12.20 g/dL [11.47–13.00] vs. 13.60 [12.60–14.70], *p*-value < 0.001, Figure 3B) were lower when compared to those in the normal group. A low vitamin D level (<20 ng/mL) was more common in the low ALT group (49.4% vs. 34.6%, *p*-value < 0.001, Figure 4). These findings remained statistically significant after application of a linear regression model controlling for age, sex, smoking status, type of IBD disease, BMI, SES, and sector (Table 2).

Amongst patients with UC, low ALT was associated with lower albumin (4.00 g/dL [3.62–4.18] vs. 4.30 [4.10–4.40], *p*-value < 0.001, Figure 3A) and lower hemoglobin (12.40 g/dL [11.60–13.20] vs. 13.60 [12.60–14.60], *p*-value < 0.001, Figure 3B). A low level of vitamin D (<20 ng/mL) was more common amongst patients in the low-ALT group (53.1% vs. 33.5%, *p*-value = 0.04), see Figure 4.

### 3.3. Propensity Score-Matching

Using a propensity score-matched algorithm, 134 subjects with IBD and low ALT were compared to 268 patients with normal ALT. In this cohort, there was no significant difference in age, gender, BMI, sector, socioeconomic status, and smoking status. In the low ALT group, fecal calprotectin, serum CRP, and platelet count were higher (205.50 g/mg [71.90–793.00] vs. 115.00 [40.70–389.00], *p*-value = 0.005, 6.90 mg/L [2.10–15.70] vs. 3.10 mg/L [1.17–8.10], *p*-value < 0.001, 306,500 U/mL [244,000–364,000] vs. 269,500 U/mL [225,000–319,250], *p*-value = 0.001, respectively). Regarding metabolic biomarkers, patients with low ALT had lower albumin (4.00 [3.69–4.20] vs. 4.20 [4.00–4.40], *p*-value < 0.001.), see Table 3. Table 4 describes the same cohort with patients only compared to those with the same disease. Despite the smaller sample size in this comparison, the results were overall still statistically significant.

## 4. Discussion

Alanine aminotransferase (ALT) is a pyridoxal enzyme that catalyzes the reversible interconversion of L-alanine and 2-oxoglutarate to pyruvate and L-glutamate. Serum ALT is generally used to assess liver health. The reported prevalence of IBD-associated hepatobiliary diseases and resulting elevated liver enzymes ranges from 3% to greater than 50% depending on the exact definition used [29]. Elevated liver enzymes can be attributed to multiple etiologies including fatty liver disease [30], drug-related liver injury [31,32,33,34], use of total parenteral nutrition (TPN) [35], systemic inflammatory processes [36], cholelithiasis [23], and primary sclerosing cholangitis [37]. Liver enzyme abnormalities were observed in adults and children with IBD [36]. In a retrospective study of 383 newly diagnosed adult patients with CD, elevated liver enzymes at diagnosis predicted a more complicated course including hospitalizations, surgeries, and mortality [24]. In a recent study by Yanai et al., serum ALT > 25 IU/L was incorporated as part of a predictive model for complicated disease in treatment naïve IBD patients [38].

Multiple studies have shown that low ALT is associated with frailty, sarcopenia, and increased mortality [13,14]. However, the prevalence and effect of low ALT in patients with IBD have not been adequately investigated. In a small study amongst pediatric patients with patients, almost half were reported to have ALT < 5 IU/L (29% at initial admission, 18% during follow-up) [26]. A Danish study performed on 127 adults with IBD found that almost all the patients with CD had subnormal ALT on at least one occasion across a 10-year follow-up. Only one patient with UC had a subnormal ALT. It should be noted that patients older than 50 years were excluded from the study, a population where low ALT is more common [27].

In this current population-based study, we showed that low ALT levels are more common in patients with IBD than in the general population. This finding remained statistically significant after controlling for multiple covariates such as age, gender, socioeconomic status, and sector. In our cohort, the overall prevalence of low ALT amongst patients with IBD was much lower than previous reports. Possible explanations for this disparity may be the shorter duration of follow-up, the more restricted definition of low ALT, and the larger cohort included in our study.

It should be noted that while other liver enzymes exist, such as aspartate aminotransferase (AST), only two patients in our IBD cohort had AST levels < 10 IU/L. This may point to ALT being a more sensitive biomarker than IBD-related inflammatory and metabolic markers.

Our study shows that amongst patients with IBD, low ALT levels was associated with increased inflammatory markers such as FC, CRP, and platelet count, and with decreased metabolic markers such as hemoglobin, albumin, and vitamin D.

Low ALT was found to be associated with a significant risk of relapse, steroid dependency, and a low level of albumin, hemoglobin, folic acid, and penetrating phenotype in a small cohort of pediatric patients with IBD [21]. Low ALT has been proposed as a surrogate marker for low muscle mass and sarcopenia [39], and patients with low ALT have a lower L3 muscle mass index [40]. In patients with IBD, multiple studies have demonstrated an increased incidence of sarcopenia amongst patients with active disease [6,41]. Thus, we can hypothesize that IBD patients’ low ALT levels in our cohort may be related to IBD activity and attributable to sarcopenia.

The most common nutrient deficiencies in IBD are of iron, vitamin B12, vitamin D, vitamin K, folic acid, selenium, zinc, vitamin B6, and vitamin B1 [34]. The ALT enzyme requires active vitamin B6 to function; thus, vitamin B6 deficiency may decrease ALT levels in inflammatory diseases, as well as in elderly patients and individuals suffering with alcohol addiction [42,43]. Nutritional deficiencies are more common in CD than in UC [44], as well as in active disease vs. remission. Thus, the higher prevalence of low ALT in CD patients in our study is consistent with this pattern. Although smoking likely lowers ALT in healthy patients and increases it in people with liver disease [45], the multivariate logistic regression model used in the current study controlled for smoking as an effecting factor.

The strength of this study is its large and heterogenous population, with data extracted from a national health insurance provider covering a diverse range of populations. Multivariate analysis and propensity score-matching methods were employed to reduce the influence of potential confounders. The results showed a consistent correlation between low ALT and inflammatory and metabolic markers.

This work has several limitations. Its retrospective nature and the absence of endoscopic evidence of IBD activity may lead to misguided conclusions. Additional research is needed to investigate whether low ALT can predict treatment failure and the frequency of flare ups. We also did not introduce any direct evidence linking low ALT with sarcopenia, for example, from imaging or muscle strength tests. Hence, this link remains a hypothesis warranting further studies.

## 5. Conclusions

In conclusion, in this large population-based cohort, low ALT, defined as ALT < 10 IU/L, is more prevalent in patients with IBD and associated with low BMI, increased inflammatory markers, and low metabolic indices. Low ALT can be used as a surrogate for disease activity and metabolic deficiencies in patients with IBD.

## Figures and Tables

**Table 1:** Comparison of demographic and medical history of the study population with low and normal ALT levels.

	ALT < 10	10 ≤ ALT < 40	*p*-Value
N	13,391	220,060	
Age (mean ± SD)	45.72 ± 22.84	46.90 ± 8.47	<0.001
Female	11,222 (83.8%)	134,462 (61.1%)	<0.001
BMI (mean ± SD)	25.62 ± 5.66	26.78 ± 5.41	<0.001
Smoking	1360 (10.2%)	29,371 (13.3%)	<0.001
Sector (%)			<0.001
Arab	2669 (19.9%)	38,413 (17.5%)	
Non Arab, Non Ultra-Orthodox	7632 (57.0%)	135,592 (61.6%)	
Ultra-Orthodox	3088 (23.1%)	45,990 (20.9%)	
Socioeconomic Status (mean ± SD)	5.03 (2.03)	5.29 (2.05)	<0.001
IBD (%)	217 (1.6%)	2580 (1.2%)	<0.001
Hypertension (%)	1432 (10.7%)	20,393 (9.3%)	<0.001
Diabetes Mellitus (%)	1715 (12.8%)	23,413 (10.6%)	<0.001
Ischemic Heart Disease (%)	837 (6.3%)	11,072 (5.0%)	<0.001

BMI—Body mass index, IBD—Inflammatory bowel disease.

**Table 2:** Comparison of demographic and laboratory parameters in IBD patients with low and normal ALT levels.

	Crohn’s Disease				Ulcerative Colitis			
	ALT < 10	10 ≤ ALT < 40	*p*-Value	*p* -Value in Multivariate Regression	ALT < 10	10 ≤ ALT < 40	*p*-Value	*p*-Value in Multivariate Regression
*n*	148	1721			69	859		
Age (mean ± SD)	39.99 ± 17.21	41.33 ± 16.20	0.337		42.43 ± 19.53	49.27 ± 17.60	0.002	
Female (%)	111 (75.0%)	898 (52.2%)	<0.001		57 (82.6%)	473 (55.1%)	<0.001	
BMI (mean ± SD)	23.35 ± 4.81	25.02 ± 5.25	0.001		24.19 ± 5.27	25.60 ± 4.70	0.041	
Smoking (%)	16 (10.8%)	250 (14.5%)	0.263		9 (13.0%)	66 (7.7%)	0.18	
Sector (%)			0.08				0.526	
Arab	8 (5.4%)	141 (8.2%)			6 (8.7%)	64 (7.5%)		
Non Arab/non Ultra-Orthodox	92 (62.2%)	1155 (67.1%)			45 (65.2%)	615 (71.6%)		
Ultra-Orthodox	48 (32.4%)	425 (24.7%)			18 (26.1%)	180 (21.0%)		
FC (median [IQR])	223.00 [63.45, 631.50]	98.50 [31.98, 324.00]	<0.001	0.009	226.50 [143.00, 537.00]	107.00 [40.85, 499.50]	0.057	0.69
FC > 150 (%)	41 (57.7)	301 (39.2)	0.004	0.016	19 (73.1)	105 (46.1)	0.016	0.015
CRP (median [IQR])	9.10 [3.22, 19.32]	3.20 [1.30, 8.30]	<0.001	<0.001	4.50 [1.90, 11.62]	2.30 [1.00, 6.20]	0.001	<0.001
Albumin (median [IQR])	3.90 [3.60, 4.20]	4.30 [4.00, 4.50]	<0.001	<0.001	4.00 [3.62, 4.18]	4.30 [4.10, 4.40]	<0.001	<0.001
Platelet (median [IQR])	295.00 [215.75, 356.00]	260.00 [216.00, 311.00]	0.003	0.001	286.00 [234.00, 342.00]	253.00 [209.00, 302.00]	0.002	0.054
Hemoglobin (median [IQR])	12.20 [11.47, 13.00]	13.60 [12.60, 14.70]	<0.001	<0.001	12.40 [11.60, 13.20]	13.60 [12.60, 14.60]	<0.001	<0.001
Vitamin D < 20 ng/mL (%)	38 (49.4)	324 (34.6)	0.014	0.012	17 (53.1)	157 (33.5)	0.04	0.22
Vitamin B12 < 280 (%)	39 (37.5)	290 (28.8)	0.082	0.016	12 (30.0)	101 (20.0)	0.192	0.29

FC—fecal calprotectin, SD—standard deviation., IQR—interquartile range.

**Table 3:** Propensity score-matched cohort.

	ALT < 10	10 ≤ ALT < 40	*p*-Value
N	134	268	
Age (mean ± SD)	38.66 (16.75)	39.38 (16.36)	0.615
Female (%)	212 (79.1%)	216 (80.6%)	0.747
BMI (mean ± SD)	23.44 (4.90)	23.65 (4.77)	0.627
Ulcerative Colitis (%)	84 (31.3%)	84 (31.3%)	1
Smoking (%)	34 (12.7%)	29 (10.8%)	0.592
Sector (%)			0.905
Arab	22 (8.2%)	23 (8.6%)	
Non Arab/non Ultra-Orthodox	170 (63.4%)	165 (61.6%)	
Ultra-Orthodox	76 (28.4%)	80 (29.9%)	
SES (mean ± SD)	5.51 (2.10)	5.45 (2.10)	0.743
CRP (median [IQR])	6.90 [2.10, 15.70]	3.00 [1.00, 8.10]	<0.001
Fecal Calprotectin (median [IQR])	205.50 [71.90, 793.00]	111.00 [40.70, 461.00]	0.006
FC > 150 (%)	86 (61.4%)	55 (44.0%)	0.007
Albumin (median [IQR])	4.00 [3.69, 4.20]	4.20 [4.00, 4.40]	<0.001
Platelets (median [IQR])	306.50 [244.00, 364.00]	265.00 [223.00, 319.00]	<0.001
Vitamin B12 (median [IQR])	327.00 [238.00, 438.75]	384.00 [282.25, 514.75]	0.003
Vitamin D < 20 ng/mL (%)	78 (52.7%)	57 (36.3%)	0.006

SES—socioeconomic status, CRP—C reactive protein. IQR—interquartile range.

**Table 4:** Propensity score-matched cohort according to specific disease.

	Ulcerative Colitis			Crohn’s Disease		
	ALT < 10	10 ≤ ALT < 40	*p*-Value	ALT < 10	10 ≤ ALT <40	*p*-Value
*n*	42	84		92	184	
Age (mean ± SD)	38.90 (19.38)	43.49 (18.55)	0.119	38.54 (15.46)	37.50 (14.94)	0.511
Female (%)	70 (83.3%)	75 (89.3%)	0.369	142 (77.2%)	141 (76.6%)	1
BMI (mean ± SD)	24.18 (5.30)	23.74 (4.22)	0.551	23.11 (4.69)	23.61 (5.02)	0.326
Smoking (%)	12 (14.3%)	3 (3.6%)	0.03	22 (12.0%)	26 (14.1)	0.642
Sector (%)			0.604			0.53
Arab	8 (9.5%)	5 (6.0%)		14 (7.6%)	18 (9.8%)	
Non Arab/non Ultra-Orthodox	52 (61.9%)	57 (67.9%)		118 (64.1%)	108 (58.7%)	
Ultra-Orthodox	24 (28.6%)	22 (26.2%)		52 (28.3%)	58 (31.5%)	
SES (mean ± SD)	5.33 (2.04)	5.83 (2.11)	0.121	5.59 (2.13)	5.27 (2.08)	0.152
CRP (median [IQR])	3.75 [1.90, 12.30]	2.65 [0.90, 6.50]	0.007	8.15 [2.82, 16.92]	3.10 [1.17, 8.72]	<0.001
Fecal Calprotectin (median [IQR])	224.00 [159.75, 734.75]	74.60 [40.70, 490.50]	0.026	193.00 [59.75, 911.50]	141.00 [41.18, 442.50]	0.074
FC > 150 (%)	30 (78.9%)	10 (32.3%)	<0.001	56 (54.9)	45 (47.9)	0.4
Albumin (median [IQR])	4.00 [3.70, 4.11]	4.20 [4.00, 4.40]	0.001	4.00 [3.67, 4.23]	4.20 [4.00, 4.40]	<0.001
Platelets (median [IQR])	302.00 [244.00, 354.00]	270.00 [218.00, 315.25]	0.024	306.50 [243.50, 365.00]	264.00 [228.00, 321.25]	<0.001
Vitamin B12 (median [IQR])	310.00 [262.00, 488.00]	413.00 [358.75, 527.50]	0.06	327.50 [224.75, 430.50]	364.50 [272.75, 501.50]	0.026
Vitamin D < 20 ng/mL (%)	22 (55.0%)	18 (39.1%)	0.209	56 (51.9%)	39 (35.1%)	0.018

## Data Availability

The datasets presented in this article are not readily available because there are ethical restrictions on sharing our data set because data contains potentially identifying patient information. These restrictions were imposed by the Ethics Committee of Meuhedet HMO who owns the data. Requests to access the datasets should be directed to Liron Yitzchaki, coordinator of Meuhedet Research Center, liron.y3@meuhedet.co.il.
